# Mildly elevated lactate levels are associated with microcirculatory flow abnormalities and increased mortality: a microSOAP post hoc analysis

**DOI:** 10.1186/s13054-017-1842-7

**Published:** 2017-10-18

**Authors:** Namkje A. R. Vellinga, E. Christiaan Boerma, Matty Koopmans, Abele Donati, Arnaldo Dubin, Nathan I. Shapiro, Rupert M. Pearse, Peter H. J. van der Voort, Arjen M. Dondorp, Tony Bafi, Michael Fries, Tulin Akarsu-Ayazoglu, Andrius Pranskunas, Steven Hollenberg, Gianmarco Balestra, Mat van Iterson, Farid Sadaka, Gary Minto, Ulku Aypar, F. Javier Hurtado, Giampaolo Martinelli, Didier Payen, Frank van Haren, Anthony Holley, Hernando Gomez, Ravindra L. Mehta, Alejandro H. Rodriguez, Carolina Ruiz, Héctor S. Canales, Jacques Duranteau, Peter E. Spronk, Shaman Jhanji, Sheena Hubble, Marialuisa Chierego, Christian Jung, Daniel Martin, Carlo Sorbara, Jan Bakker, Can Ince, for the microSOAP study group, E. C. Boerma, M. Koopmans, N. A. R. Vellinga, M. van Iterson, P. H. J. van der Voort, J. Bakker, P. E. Spronk, C. Ruiz, A. T. Bafi, A. Dubin, V. S. Kanoore Edul, H. S. Canales, F. J. Hurtado, S. M. Hollenberg, N. I. Shapiro, H. Gomez, F. G. Sadaka, R. Mehta, C. Jung, M. Fries, R. M. Pearse, D. S. Martin, P. Meale, S. Jhanji, G. Minto, G. Martinelli, M. Lombrano, S. M. A. Hubble, A. H. Rodriguez, I. Martin-Loeches, F. M. P. van Haren, A. Pranskunas, V. Pilvinis, A. Donati, C. Sorbara, M. L. Chierego, A. Holley, J. Duranteau, A. Harrois, D. Payen, G. M. Balestra, A. M. Dondorp, U. Aypar, J. van Bommel, G. Hernandez, F. Machado, V. Kanoore Edul, G. Lacuesta, M. Baz, U. Patel, M. Pinsky, K. Krause, A. Smith, C. Thorn, A. Forti, A. Comin, T. Pellis, J. Paratz, C. Damoisel, E. Bucher, R. Pattnaik, M. T. Herdman, B. Ayhan

**Affiliations:** 1 Department of Intensive Care Adults, Erasmus MC University Medical Center, Rotterdam, The Netherlands; 2 Department of Intensive Care, Medical Center Leeuwarden, P.O. Box 888, 8901 BR Leeuwarden, The Netherlands; 3 Department of Biomedical Science and Public Health, Università Politecnica delle Marche, Ancona, Italy; 4 Sanatorio Otamendi y Miroli, Servicio de Terapia Intensiva, Azcuénaga 870, Buenos Aires, Argentina; 5 Department of Emergency Medicine and Center for Vascular Biology Research, Beth Israel Deaconess Medical Center, Boston, MA USA; 6 Barts and The London School of Medicine and Dentistry, London, UK; 7 Department of Intensive Care, Onze Lieve Vrouwe Gasthuis, Amsterdam, The Netherlands; 8 Faculty of Tropical Medicine, Mahidol University, Bangkok, Thailand; 9 Dor e Terapia Intensiva, Universidade Federal de São Paolo, São Paolo, Brasil; 10 Department of Anesthesia and Surgical Intensive Care, St. Vincenz Krankenhaus, Limburg, Germany; 11 S.B. Medeniyet University Göztepe Education and Research Hospital Kadıköy, Istanbul, Turkey; 12 Intensive Care Department, Lithuanian University of Health Sciences, Kaunas, Lithuania; 13 Section of Cardiology, Cooper University Hospital, Camden, NJ USA; 14 Medical Intensive Care Unit, University Hospital Basel, Basel, Switzerland; 15 Department of Anesthesiology, Intensive Care and Pain Management, St. Antonius Hospital, Nieuwegein, The Netherlands; 16 Critical Care Medicine/Neurocritical Care, Mercy Hospital St. Louis, St. Louis University Hospital, St. Louis, MO USA; 17 Derriford Hospital, Plymouth University Peninsula School of Medicine, Plymouth, UK; 18 Intensive Care Unit, Hacettepe University, Ankara, Turkey; 19 Intensive Care Unit, Hospital Español-State Health Administration Service, School of Medicine, University of the Republic, Montevideo, Uruguay; 20 Department of Perioperative Medicine, Barts Heart Centre, St. Bartholomew’s Hospital, London, UK; 21 Department of Anesthesiology, Critical Care and Mobile Emergency and Resuscitation Service (SMUR), Hôpital Lariboisière Assistance Publique – Hôpitaux de Paris (AP-HP)/Université Paris 7 Diderot, Paris, France; 22 Intensive Care Unit, Canberra Hospital, Canberra, Australia; 23 Department of Intensive Care Medicine, Royal Brisbane & Women’s Hospital, Brisbane, Australia; 24 Critical Care Medicine, University of Pittsburgh, Pittsburgh, PA USA; 25 School of Medicine, University of California, San Diego, San Diego, CA USA; 26 Critical Care Department, Joan XXIII University Hospital, Tarragona, Spain; 27 Departamento de Medicina Intensiva, Escuela de Medicina, Facultad de Medicina, Universidad Católica de Chile, Santiago, Chile; 28 Intensive Care Unit, Hospital San Martín, La Plata, Argentina; 29 Departement d’Anesthesie-Reanimation, Hôpitaux Universitaires Paris-Sud, Université Paris-Sud, Hôpital de Bicêtre Assistance Publique – Hôpitaux de Paris (AP-HP), Le Kremlin-Bicêtre, Paris, France; 30 Intensive Care Unit, Gelre Ziekenhuizen, Apeldoorn, The Netherlands; 31 Intensive Care Unit, The Royal Marsden Hospital, London, UK; 32 Intensive Care Unit, Royal Devon and Exeter Hospital, Exeter, UK; 33 Intensive Care Unit, Santa Maria degli Angeli Hospital, Pordenone, Italy; 34 Department of Cardiology, Universitätsherzzentrum Thüringen, Clinic of Internal Medicine I, Friedrich Schiller University Jena, Jena, Germany; 35 Division of Cardiology, Pulmonology, and Vascular Medicine, Medical Faculty, University Düsseldorf, Düsseldorf, Germany; 36 Intensive Care Unit, Royal Free Hospital, London, UK; 37 Dipartimento di Anestesia, Rianimazione e Terapia Intensiva, Azienda Unità Locale Socio Sanitaria 9 (ULSS 9) Veneto, Treviso, Italy

**Keywords:** Lactate, Microcirculation, SDF imaging, Intensive care, Prevalence

## Abstract

**Background:**

Mildly elevated lactate levels (i.e., 1–2 mmol/L) are increasingly recognized as a prognostic finding in critically ill patients. One of several possible underlying mechanisms, microcirculatory dysfunction, can be assessed at the bedside using sublingual direct in vivo microscopy. We aimed to evaluate the association between relative hyperlactatemia, microcirculatory flow, and outcome.

**Methods:**

This study was a predefined subanalysis of a multicenter international point prevalence study on microcirculatory flow abnormalities, the Microcirculatory Shock Occurrence in Acutely ill Patients (microSOAP). Microcirculatory flow abnormalities were assessed with sidestream dark-field imaging. Abnormal microcirculatory flow was defined as a microvascular flow index (MFI) < 2.6. MFI is a semiquantitative score ranging from 0 (no flow) to 3 (continuous flow). Associations between microcirculatory flow abnormalities, single-spot lactate measurements, and outcome were analyzed.

**Results:**

In 338 of 501 patients, lactate levels were available. For this substudy, all 257 patients with lactate levels ≤ 2 mmol/L (median [IQR] 1.04 [0.80–1.40] mmol/L) were included. Crude ICU mortality increased with each lactate quartile. In a multivariable analysis, a lactate level > 1.5 mmol/L was independently associated with a MFI < 2.6 (OR 2.5, 95% CI 1.1–5.7, *P* = 0.027).

**Conclusions:**

In a heterogeneous ICU population, a single-spot mildly elevated lactate level (even within the reference range) was independently associated with increased mortality and microvascular flow abnormalities. In vivo microscopy of the microcirculation may be helpful in discriminating between flow- and non-flow-related causes of mildly elevated lactate levels.

**Trial registration:**

ClinicalTrials.gov, NCT01179243. Registered on August 3, 2010.

**Electronic supplementary material:**

The online version of this article (doi:10.1186/s13054-017-1842-7) contains supplementary material, which is available to authorized users.

## Background

An elevated lactate level, classically defined as an arterial lactate level > 2 mmol/L, is a well-known predictor of adverse outcome in terms of organ dysfunction and mortality in different subgroups of critically ill patients [1–3]. Surviving Sepsis Campaign guidelines consider a threshold of 1 mmol/L as an indicator of tissue hypoperfusion, but they suggest resuscitation to normalize arterial lactate levels in patients with lactate levels > 4 mmol/L in order to improve outcome, based on the principles of early goal-directed therapy [4–6]. Similarly, in nonseptic patients, the value of lactate levels in goal-directed resuscitation, as well as the additive value of serial lactate measurements, is recognized [7–10]. Recent studies indicate that small increases in lactate levels are already associated with an unfavorable clinical course. This association has been demonstrated for “relative hyperlactatemia” with thresholds as low as 1.1 mmol/L [11–14]. Although lactate is easily measured in daily practice, unraveling the underlying causative mechanism is often much more difficult. Organ hypoperfusion is regarded as an important cause of hyperlactatemia, although several other mechanisms also play a significant role, ranging from accelerated aerobic glycolysis to decreased lactate metabolism and mitochondrial and microcirculatory dysfunction [15]. Sublingual direct in vivo microscopy is a suitable method of detecting microcirculatory derangements at the bedside [16]. Several studies have demonstrated an association between lactate levels and microcirculatory alterations in subgroups of critically ill patients as well as in experimental settings [17–25]. We previously demonstrated that both microcirculatory derangements and arterial lactate levels were independent predictors of mortality in selected high-risk patients [26].

The aforementioned studies were primarily focused on the early phase of intensive care unit (ICU) admission. The significance of minimally elevated lactate levels as well as concomitant microcirculatory dysfunction at a later time point is unclear. Therefore, we aimed to investigate the significance of a single-spot arterial lactate measurement and simultaneous in vivo microscopy in a heterogeneous ICU population recruited from 36 ICUs worldwide.

## Methods

### Patients and setting

This study was a post hoc analysis of a prospective observational point prevalence study of the prevalence and significance of microcirculatory alterations in a heterogeneous ICU population (Microcirculatory Shock Occurrence in Acutely ill Patients [microSOAP; ClinicalTrials.gov identifier NCT01179243; registered on August 3, 2010 [26]). Thirty-six ICUs worldwide participated in this study. Being a point prevalence study, data collection on patient characteristics and laboratory values, as well as simultaneous sublingual sidestream dark-field (SDF) imaging, was performed on a single day for all patients in a given ICU or ICU subunit. Lactate levels were measured within a maximum of 4 h before or after SDF imaging. For this substudy, patients with an arterial lactate level ≤ 2 mmol/L were included.

### Ethics approval

Every participating center obtained ethics approval according to local legislation. A copy of the ethics approval was sent to the study coordinator before the start of the study (*see* Additional file 1). Written informed consent was obtained from all included subjects, unless the local ethics committee specifically allowed a waiver in this respect.

### SDF imaging

SDF imaging is a noninvasive technique consisting of a camera incorporated in a handheld device that emits stroboscopic green light with a wavelength within the absorption spectrum of hemoglobin (Hb) [12]. The light emitted by the SDF camera (MicroScan; MicroVision Medical, Amsterdam, The Netherlands) is absorbed by Hb, visualizing erythrocytes as black cells on the screen. Offline software-assisted analysis of SDF images (AVA 3.0; MicroVision Medical) yields information on convective oxygen transport and diffusion distance. The semiquantitative microvascular flow index (MFI), ranging from 0 (no flow) to 3 (continuous flow), and the percentage of perfused vessels (PPV) provides information on convection, whereas total vessel density (TVD) and perfused vessel density (PVD) provide information on diffusion [27]. A single measurement consisting of three sublingual SDF image sequences of 10–20 seconds was obtained for every patient. SDF imaging as well as subsequent image analysis were performed in line with international consensus [27, 28].

### Statistical analysis

Analysis was focused on associations between lactate levels, mortality, organ dysfunction, and microcirculatory alterations. An abnormal microcirculatory blood flow was predefined as a sublingual MFI < 2.6 for vessels < 20 μm, being the lowest reported lower bound of the 95% CI of healthy volunteers. We defined this value a priori for the analysis of the original microSOAP data. A post hoc analysis confirmed this cutoff value as the Youden index in an ROC curve [26]. This cutoff value has been validated as clinically relevant [26, 29]. To determine cutoff values for lactate levels for both abnormal MFI and mortality, the AUC was calculated. These cutoff values were subsequently tested in logistic regression analysis.

Backward stepwise logistic regression was employed to detect determinants of a capillary MFI < 2.6. Predictors with *P* < 0.25 in univariable logistic regression were used for multivariable modeling (*see* Additional file 2 for additional information on the statistical analysis). Tested predictors included Sequential Organ Failure Assessment (SOFA) score on the day of SDF imaging, Acute Physiology and Chronic Health Evaluation II (APACHE II) score on ICU admission, length of stay in the ICU prior to SDF imaging (≤24 h and > 24 h), admission diagnosis, the presence of sepsis at the time of SDF imaging, Hb ≤ 5.37 mmol/L, arterial lactate level > 1.5 mmol/L, heart rate, mean arterial pressure, fluid balance, and vasopressor use. In case of nonlinearity of the logit, variables were dichotomized. The resulting models were tested for multicollinearity. Hosmer and Lemeshow goodness of fit was used to test the fit of the model. Furthermore, the associations between lactate levels, microcirculatory dysfunction, mortality, and organ dysfunction (SOFA, cumulative vasopressor index [CVI] [30]) were described by dividing the lactate measurements into quartiles. To test for differences between normally distributed variables, Student’s *t* test or the Mann-Whitney *U* test was performed. To compare dichotomous variables, Fisher’s exact test was applied. Distributions across more than two groups were tested using the nonparametric Kruskal-Wallis test. The data were analyzed using IBM SPSS Statistics version 23.0 (IBM, Armonk, NY, USA) and Prism 5.04 (GraphPad Software, Inc., La Jolla, CA, USA) software and are presented as the median [IQR] or mean ± SD, unless indicated otherwise. *P* < 0.05 was considered statistically significant.

## Results

### General characteristics

Out of 501 patients, arterial lactate levels were available for 338 (67%) of patients. In 257 out of these 338 patients (76%), arterial lactate levels were ≤ 2 mmol/L. These patients, with median APACHE of 16 [10–23] and median SOFA of 5 [3–8], were included for further analysis (Table 1). Surgery (35.4%) and sepsis (17.5%) were the main reasons for ICU admission. Median arterial lactate levels were 1.04 [0.80–1.40] mmol/L. ICU and hospital mortality were 20.6% and 27.2%, respectively.

**Table 1:** Patient characteristics

Characteristics	Data
Age, years	64 [52–74]
Male sex, *n* (%)	156 (61)
APACHE II score^a^	16 [10–23]
SOFA score^b^	5 [3–8]
ICU mortality, *n* (%)	53 (20.6)
In-hospital mortality, *n* (%)	70 (27.2)
Time in ICU before SDF imaging, days	4.0 [0.8–9.0]
<24 h in ICU before SDF imaging, *n* (%)	79 (30.7)
Reason for ICU admission, *n* (%)	
Surgery	91 (35.4)
Sepsis	45 (17.5)
Cardiac disease	18 (7.0)
Neurological disorders	27 (10.5)
Trauma	30 (11.7)
Respiratory insufficiency	21 (8.2)
Other	25 (9.7)
Arterial lactate, mmol/L	1.04 [0.80–1.40]
Hemoglobin, mmol/L	6.2 [5.4–7.0]
Vasopressor drugs	
Patients treated, *n* (%)	89 (34.6)
Cumulative vasopressor index^c^	3 [2–4]
Mechanical ventilation, *n* (%)	161 (63)
Abnormal microcirculation^d^, *n* (%)	36 (14)
MFI small vessels, AU	2.9 [2.7–3.0]
MFI large vessels, AU	3.0 [2.9–3.0]
TVD, mm/mm^2^ (small vessels)	18.9 [15.7–21.2]
PVD, mm/mm^2^ (small vessels)	18.1 [15.0–20.6]
PPV, % (small vessels)	98 [95–99]
De Backer score (small vessels)	11.3 ± 2.5
De Backer score (perfused small vessels)	10.9 ± 2.4
Heterogeneity index (small vessels)	0.07 [0.00–0.25]

*Abbreviations: APACHE II* Acute Physiology and Chronic Health Evaluation II, *ICU* Intensive care unit, *MFI* Microvascular flow index, *PPV* Percentage of perfused vessels, *PVD* Perfused vessel density, *SDF* Sidestream dark-field imaging, *SOFA* Sequential Organ Failure Assessment, *TVD* Total vessel density

Values are mean ± SD or median [IQR] unless specified otherwise. Cutoff value for small vessels < 20 μm

^a^APACHE II scores range from 0 to 71, with higher values indicating more severe disease

^b^SOFA scores range from 0 to 4 for each organ system, with higher scores indicating more severe organ dysfunction

^c^Trzeciak et al. [30]

^d^Abnormal microcirculation defined as small vessel MFI < 2.6

### Lactate levels and mortality

**Fig. 1:**
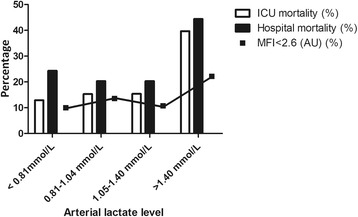
Arterial lactate levels (quartiles) and distribution of intensive care unit (ICU)/hospital mortality and abnormal microvascular flow index (MFI < 2.6). *P* < 0.001 for ICU mortality, *P* = 0.005 for hospital mortality, *P* = 0.169 for abnormal MFI for distributions over quartiles

Increases in ICU mortality were observed for every lactate quartile (≤0.80 mmol/L, 12.9%; 0.81–1.04 mmol/L, 15.3%; 1.05–1.40 mmol/L, 15.4%; > 1.40 mmol/L, 39.7%; *P* < 0.001). Similar trends were observed for hospital mortality (24.3% in the lowest quartile, 44.4% in the highest quartile; *P* = 0.005) (*see* Fig. 1). The AUC was 0.65 (95% CI 0.56–0.73, *P* = 0.001) with a cutoff value of 1.42 mmol/L for ICU mortality (sensitivity 40%, specificity 81%). The same cutoff value was seen for hospital mortality with a sensitivity of 47% and a specificity of 81% (AUC 0.59, 95% CI 0.51–0.67, *P* = 0.025). Mortality was at least almost twice as high for patients with an arterial lactate level > 1.5 mmol/L as compared with patients with a lower lactate level (ICU mortality 41.2% vs. 15.5%, *P* < 0.001; hospital mortality 45.1% vs. 22.9%, *P* = 0.001).

### Lactate levels and microcirculatory flow abnormalities

Patients with a capillary MFI < 2.6 (14%) had slightly but nonsignificantly higher lactate levels than patients with a higher MFI (1.11 [0.90–1.60] vs. 1.00 [0.80–1.40] mmol/L, *P* = 0.117). A nonsignificant trend toward a higher prevalence of an abnormal microcirculation in the highest lactate quartile was observed (*P* = 0.169) (Fig. 1). Hb was significantly lower in patients with an MFI < 2.6 (Hb 5.4 [5.2–6.8] vs. Hb 6.3 [5.5–7.1], *P* = 0.011). No significant differences with respect to illness severity scores, hemodynamics, vasopressor use or dose, reason for ICU admission, or time in ICU prior to SDF imaging were observed. Comparing patients with lactate levels ≤ 1.5 mmol/L and > 1.5 mmol/L, no significant differences were observed for small vessel MFI; large vessel MFI; and small vessel TVD, PVD, PPV, (perfused) De Backer score, and heterogeneity index.

### Multivariable logistic regression analysis for MFI < 2.6

In multivariable logistic regression analysis, the only remaining significant predictors for an abnormal MFI were an Hb ≤ 5.37 mmol/L (OR 4.6, 95% CI 2.1–10.2; *P* < 0.001), a stay in the ICU < 24 h prior to SDF (OR 2.9, 95% CI 1.3–6.6, *P* = 0.008), and an arterial lactate level > 1.5 mmol/L (OR 2.5, 95% CI 1.1–5.7, *P* = 0.027). The AUC for this three-variable model was 0.74 (95% CI 0.65–0.83, *P* = 0.001). The Hosmer and Lemeshow chi-square statistic was 2.015 (*P* =0.847) (*see also* Additional file 2).

### Lactate levels and organ dysfunction

**Fig. 2:**
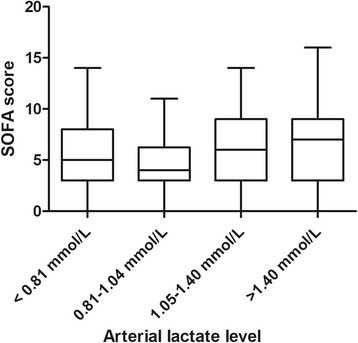
Sequential Organ Failure Assessment (SOFA) scores per arterial lactate quartile. *P* = 0.078 for distributions over quartiles

**Fig. 3:**
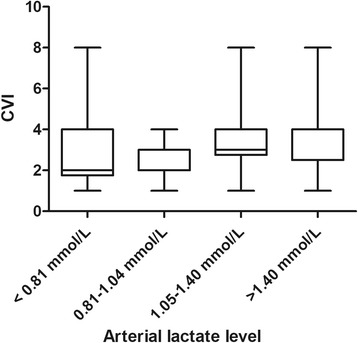
Cumulative vasopressor index (CVI) per arterial lactate quartile. *P* = 0.063 for distributions over quartiles

A higher lactate level was not accompanied by a significantly higher SOFA score or CVI (*P* = 0.078 and *P* = 0.063, respectively) (Figs. 2 and 3).

### Different phenotypes

**Fig. 4:**
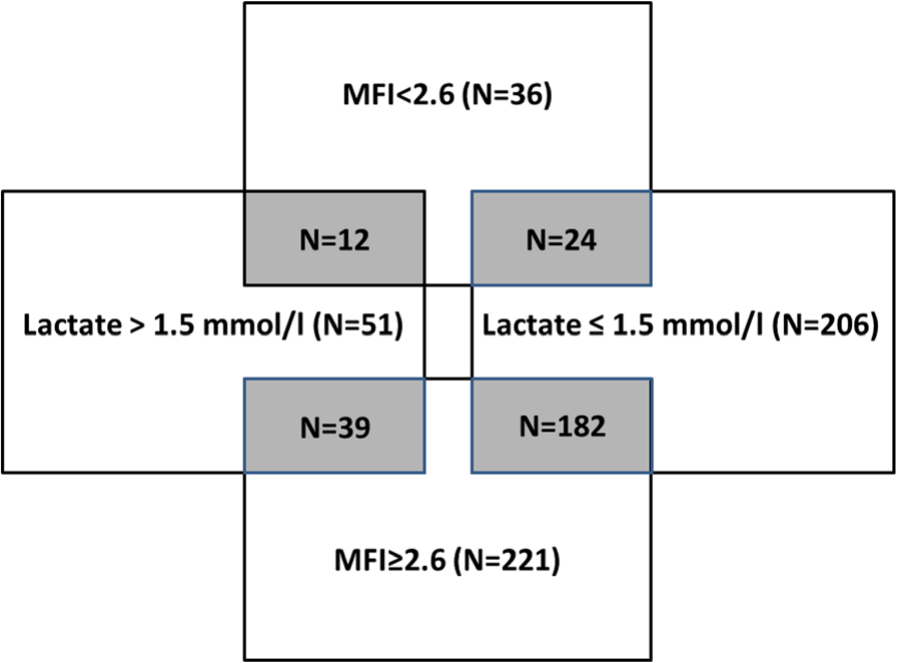
Venn diagram depicting overlap between an abnormal microcirculation and lactate groups. *MFI* Microvascular flow index (AU)

Although an abnormal MFI and elevated lactate levels appear to be associated, several different phenotypes exist. For individual patients, a higher lactate level was not necessarily associated with adverse outcome or an abnormal microcirculation or vice versa, pointing toward a multifactorial etiology and significance of both hyperlactatemia and microvascular derangements (Fig. 4).

## Discussion

In the present study, a single-spot arterial lactate level > 1.5 mmol/L was associated with increased mortality as well as with microcirculatory abnormalities and organ dysfunction. This “relative hyperlactatemia” is an emerging concept [11–14, 31]. Lactate levels on admission as low as 1.1 mmol/L already appeared to be associated with adverse outcome [12]. Our observations add to the idea that the prognostic relevance of mildly elevated lactate levels is not restricted to the early phase of ICU admission. The twofold increase in mortality in patients with a lactate level > 1.5 mmol/L is in agreement with results of previous studies focused on the first day of ICU admission [13, 14]. Researchers in a few studies have reported lactate levels and their association with outcome during the later phase of ICU stay, showing contradictory results. Some have observed an association between hyperlactatemia after initial stabilization with higher mortality rates, whereas others found that not lactate itself but impaired lactate clearance was associated with adverse outcome [32, 33]. In this respect, it is notable that we were able to demonstrate this association in a highly heterogeneous study population, in terms of both the timing of the lactate measurement as well as the underlying diagnosis and disease severity. Not only mortality but also organ dysfunction in terms of SOFA score appeared to be more severe for increasing lactate levels, albeit that this was statistically nonsignificant. A previous study was able to show associations between incremental lactate levels > 2 mmol/L and SOFA scores [2]. However, in that study, the investigators evaluated the time course of lactate measurements, whereas in the present study, we evaluated the implications of a single lactate measurement.

Several mechanisms may be involved in the increase of lactate levels. One of these, microcirculatory flow abnormalities, was indeed associated with mildly elevated lactate levels in the present study. PVD, and therefore effective diffusion distance, did not differ between patients with and without mildly elevated lactate levels. Therefore, impaired convective oxygen transport, but not diffusion distance, might have contributed to anaerobic glycolysis. Several researchers have also observed an association between impairment of microvascular flow and elevations in arterial lactate, whereas others have been able to demonstrate associations between lactate levels and parameters of vessel density in a variety of disease states [17, 19–25].

Alternatively, several non-flow-related factors may lead to increased nonanaerobic lactate formation under conditions of stress by promoting conversion of glucose to lactate via pyruvate instead of pyruvate entering the citric acid cycle [15, 34]. Indeed, lactate formation in endotoxemia results predominantly from increased aerobic lactate formation [35]. On top of that, exogenous adrenergic stress resulting from β-adrenergic drugs can also increase aerobic lactate formation [36].

Besides ongoing lactate formation, impaired lactate clearance has to be kept in mind as a cause of mildly elevated lactate levels. Levraut and coworkers observed that in stable septic patients in whom arterial lactate levels were < 2 mmol/L after the initial resuscitation phase, impaired clearance of exogenous sodium lactate but not baseline lactate values could discriminate between survivors and nonsurvivors [32, 37]. It is conceivable that a similar mechanism was involved in our patients.

Altogether, the direct observation of the microcirculation in conjunction with lactate measurements confirms the idea that impaired organ perfusion is only one of many explanations for elevated lactate levels with potential consequences for therapeutic strategies in the ICU [29, 38].

Our study has several limitations. At first glance, the absolute numbers of lactate and MFI seem to indicate that the study population was not severely ill. However, owing to the design of the study, patients with a longer stay in the ICU before study inclusion were overrepresented. Therefore, the median APACHE II score of 16 seems to be a better indicator of considerable severity of illness of the population at ICU admission. The lack of macrohemodynamic monitoring limited in-depth statistical analysis of factors associated with relative hyperlactatemia. Furthermore, no detailed information on factors influencing lactate clearance or drugs influencing lactate metabolism (e.g., metformin) was available. In addition, the presence of microvascular flow abnormalities in other organs not detected by sublingual in vivo microscopy cannot be ruled out [39]. Serial measurements of both microcirculation and lactate could have shed more light on the time course of organ dysfunction in patients with relative hyperlactatemia [30, 40, 41]. Although independently associated in the multivariate analysis, it is conceivable that a factor not accounted for in our model influenced both lactate and MFI. Last, it should be stated that the observed association between relatively low lactate levels and outcome does not automatically imply clinical relevance. Not only is the predictive value of this multivariate model relatively low with an AUC of 0.74, but it also remains to be established whether interventions aimed at achieving a further reduction of lactate will be beneficial to patients.

## Conclusions

Our data indicate that even single-spot lactate levels within the usual reference range are associated with an unfavorable clinical course. However, the question remains how the clinician must incorporate these findings into an individualized approach to treating otherwise seemingly stable ICU patients. In vivo microscopy of the (sublingual) microcirculation may be helpful for detection of organ perfusion-related causes of mildly elevated lactate levels with potential consequences for a therapeutic strategy.

## Additional files

Additional file 1: Declarations of ethical approval. (DOCX 23 kb)

Additional file 2: Supplemental material. (DOCX 14 kb)

## Abbreviations

APACHE II: Acute Physiology and Chronic Health Evaluation II
CVI: Cumulative vasopressor index
Hb: Hemoglobin
ICU: Intensive care unit
MFI: Microvascular flow index
microSOAP: Microcirculatory Shock Occurrence in Acutely ill Patients
PPV: Percentage of perfused vessels
PVD: Perfused vessel density
SDF: Sidestream dark-field imaging
SOFA: Sequential Organ Failure Assessment
TVD: Total vessel density
