# Supplementary material for: Mildly elevated lactate levels are associated with microcirculatory flow abnormalities and increased mortality: a microSOAP post hoc analysis
Source: Crit Care. 2017 Oct 18;21:255. doi: 10.1186/s13054-017-1842-7 (PMC5646128; doi:10.1186/s13054-017-1842-7)
Supplement: Supplementary file 1 — Declarations of ethical approval. (DOCX 23 kb) [file 13054_2017_1842_MOESM1_ESM.docx]

**Additional file 1**

**Overview of Medical Ethics Commmittees**

| **Center** | **Medical ethics committee** | **Ref.** |
| --- | --- | --- |
| ICU, Medical Center Leeuwarden, the Netherlands | Regionale Toetsingscommissie Patiëntgebonden Onderzoek, Leeuwarden NL | NL 31331.099.10 |
| ICU, Antonius Ziekenhuis, Nieuwegein, the Netherlands | Regionale Toetsingscommissie Patiëntgebonden Onderzoek, Leeuwarden NL | NL 31331.099.10 |
| ICU, Onze Lieve Vrouwe Gasthuis, Amsterdam, the Netherlands | Regionale Toetsingscommissie Patiëntgebonden Onderzoek, Leeuwarden NL | NL 31331.099.10 |
| ICU, Erasmus Medical Center, Rotterdam, the Netherlands | Regionale Toetsingscommissie Patiëntgebonden Onderzoek, Leeuwarden NL | NL 31331.099.10 |
| ICU, Gelre Ziekenhuizen, Apeldoorn, the Netherlands | Regionale Toetsingscommissie Patiëntgebonden Onderzoek, Leeuwarden NL | NL 31331.099.10 |
| Hospital Clinico de la Pontificia Universidad Católica de Chile. Departamento de Medicina Intensiva, Santiago, Chili | Pontificia Universidad Catolica de Chile, Facultad de Medicina, Escuela de Medicina - Ccomité de Etica en Investigación | 11-112 |
| ICU, Hospital Sao Paulo, Sao Paulo, Brasil | Conselho Nacional de Saúde, Comissão Nacional de ética em pesquisa | CONEP 16573 |
| Sanatorio Otamendi y Miroli, Servicio de Terapia Intensiva, Buenos Aires, Argentina | Comité de Ethica en Investigacines Biomedicas Sanatorio Otamendi y Miroli S.A. | - |
| Hospital San Martín, La Plata, Argentina | H.I.G.A. San Martín de La Plata, Comité de Ética de la Investigación, Comité de Bioética | - |
| ICU, Hospital Español 'Juan J Crotoggini', Montevideo Uruguay | Comisión de Bioética del Hospital Español Dr. JJ Crottogini | - |
| ICU, Cooper University Hospital, Camden, USA | The Cooper Health System Institutional Review Board | 11-127 EX |
| Beth Israel Deaconess Medical Center/Harvard Medical School, Boston USA | Committee on Clinial Investigations | 2011-P-000161; BIDMC |
| Critical Care Medicine, University of Pittsburgh, Pittsburgh, PA, USA | University of Pittsburgh, Institutional Review Board | [REN11030276 / PRO08120096](https://www.osiris.pitt.edu/osiris/Rooms/DisplayPages/LayoutInitial?Container=com.webridge.entity.Entity%5bOID%5b92BCC6C4FC2E9E478E33BFEB789ED5F5%5d%5d) |
| Critical Care Medicine, St. John’s Mercy Medical Center, St Louis, Missouri, USA | St. John's Mercy Medical Center Institutional Review Board | 11-046 |
| University of California, San Diego, USA | University of California, San Diego, Human Research Protections Program | 111230 |
| Universitätsklinikum Jena, Friedrich-Schiller-University, Department of Internal Medicine I, Jena Germany | Universitätsklinikum Jena, Ethik-Kommission | 11-5-3134 |
| ICU, University Hospital Aachen, Germany | Ethik-Kommission an der medizinische Fakultät der Rheinisch-Westfälischen technischen Hochschule Aachen | EK 225/11 |
| ICU, Royal London Hospital, London, UK | Barts and the London Joint Research and Development Office | 8001 |
| ICU,(UCL), Royal Free Hospital, London, UK | National Research Ethics Service (including general approval for all UK NHS sites participating in the microSOAP study) | 11/YH/0273 |
| The Royal Marsden Hospital,Chelsea, London, UK | Royal Marsden Committee for Clinical Research | CCR3714 |
| ICU, Derriford Hospital and Plymouth Hospital, Plymouth, UK | Plymuth Hospitals NHS Trust Research & Development Office | 11/P/058 |
| ICU, New Cross Hospital, Wolverhampton, UK | The Royal Wolverhampton Hospitals Research & Development Directorate | 11CRIT03 |
| RDE Hospital, Exeter, UK | Royal Devon and Exeter NHS Foundation Trust | 1205643 |
| Critical Care Department, Joan XXIII University Hospital, Tarragona, Spain | Hospita; Universitari de Tarragona, Joan XXIII, Comité Etico de Investigacion Clinica | NTC 1179243 |
| Waikato Hospital; Department of Intensive Care Medicine, Hamilton, New Zealand | Northern Y Regional Ethics Committee | NTY/11/08/087 |
| Kaunas University Hospital, Kaunas, Litouwen | Ethic commission Kaunas University |  |
| Azienda Ospedaliera-Universitaria Ospedali Riuniti, Clinica di Anestesia e Rianimazione, Ancona, Italy | Comitato Etico dell'Azienda Ospedaliero Universitaria Ospedali Riuniti Umberto I- G.M. Lancisi- G. Salesi di Ancona | N.211312 |
| Dipartimento di Anestesia, Rianimazione e Terapia Intensiva Azienda ULSS 9 Veneto, Treviso, Italy | Regione Veneto Comitato Etico Provinciale per la sperimentazione | 327/AULSS9 |
| Pordenone, Italy | Comitato Etico Indipendente | 29 |
| ICU, Royal Brisbane and Women's Hospital, Brisbane, Australia |  |  |
| Departement d'Anesthesie-Reanimation, Hopital de Bicetre Le Kremlin- Bicêtre, Paris, France |  |  |
| ICU, Hôpital Lariboisière, Paris | waiver (Prof E. Vicaut, Unite Recherche Clinique) | - |
| ICU, University Hospital Basel, Switzerland | Ethikkommission beider Basel | 254/11 |
| Faculty of Tropical Medicine, Mahidol University, Bangkok, Thailand | Institutional Review Board Ispat General Hospital Rourkela, Orissa | - |
| Hacettepe University, Ankara, Turkey | Hacettepe University Faculty of Medicine Department of Anesthesiology and Reanimation | HEK 10/72-6 |
| K. Kosuyolu High Specialty Education and Research Hospital, Kartal Kosuyolu, Kosuyolu University, Istanbul, Turkey | Yeditepe Universitesi Tip Fakültesi Klinik Arastirmalar Degerlendirme | 142 |
