# Supplementary material for: Mildly elevated lactate levels are associated with microcirculatory flow abnormalities and increased mortality: a microSOAP post hoc analysis
Source: Crit Care. 2017 Oct 18;21:255. doi: 10.1186/s13054-017-1842-7 (PMC5646128; doi:10.1186/s13054-017-1842-7)
Supplement: Supplementary file 2 — Supplemental material. (DOCX 14 kb) [file 13054_2017_1842_MOESM2_ESM.docx]

**Supplemental material to “Mildly elevated lactate levels are associated with microcirculatory flow abnormalities, organ dysfunction and increased mortality: a microSOAP post hoc analysis”**

As described in the Methods Section, multivariable logistic regression was applied to detect associations between an abnormal MFI (<2.6) and several variables.

Both stay in ICU before SDF imaging, hemoglobin and cumulative vasopressor use were dichotomized because of non linear associations of risk levels and outcome

- Stay in ICU before SDF imaging was divided into ≤ 24 hours and > 24 hours
- Cumulative vasopressor use was divided into “vasopressor use” and “no vasopressor use”
- Hemoglobin ≤ 5.37 mmol/l and > 5.37 mmol/l (i.e. lowest quartile vs. other quartiles). Because hemoglobin was not available in 8 out of 257 patients, a dummy variable was included in the multivariable analysis to account for the potential effect of a missing hemoglobin value.

For admission diagnosis, data were re-grouped because the seven groups as depicted in table 1 (baseline characteristics) were considered too fragmented for inclusion in multivariable analysis.
Admission diagnosis was regrouped as 1) sepsis, 2) trauma/haemorrhage/other, 3) respiratory/cardiac disease, 4) neurological disorders.

These dichotomization (except for hemoglobin) and the regrouping of the admission diagnosis was the same as was used in the statistical analysis for the first article on the microSOAP results (Crit Care Med 2015;43(1):48-56).

Results of the univariable logistic regression with MFI<2.6 as outcome variable:
SOFA score on the day of SDF imaging P = 0.917
APACHE II on the day of ICU admission P = 0.243
Length of stay in the ICU ≤ 24 hours prior to SDF imaging P = 0.058
Sepsis at the time of SDF imaging P = 1.000
Admission diagnosis, overall effect P = 0.644
Hemoglobin ≤ 5.37 mmol/l P = 0.001
Arterial lactate > 1.5 mEq/L P = 0.032
Heart rate P = 0.367
Mean arterial pressure P = 0.311
Fluid balance P = 0.996
Vasopressor use P = 0.353

The variables mentioned above with a P<0.25 in univariable analysis were included in backwards stepwise modelling. With every step, the variable with the highest P-value was omitted until all variables in the model had P-values below 0.05. As a double check, the analysis was repeated after omitting the 8 patients without hemoglobin available. This yielded the same multivariable model, with roughly the same P-values and Odds ratios.
